# An Antiseptic Vaginal Tampon

**Published:** 1904-12

**Authors:** 


### AN ANTISEPTIC VAGINAL TAMPON.

In cases where inflammatory conditions call for the use of a medicated tampon, the following will be found most satisfactory and free from danger or objections:

℞ Pulv. Alum.................................. 30 grams.
Hux-Sal ....................................... 2 grams.
Glycerini ....................................300 grams.
Aquae ........................................200 grams.
Misce.

Sig. Use to moisten vaginal tampons.

